# Biochemical Characteristics of iPSC-Derived Dopaminergic Neurons from N370S *GBA* Variant Carriers with and without Parkinson’s Disease

**DOI:** 10.3390/ijms24054437

**Published:** 2023-02-23

**Authors:** Elena V. Grigor’eva, Alena E. Kopytova, Elena S. Yarkova, Sophia V. Pavlova, Diana A. Sorogina, Anastasia A. Malakhova, Tuyana B. Malankhanova, Galina V. Baydakova, Ekaterina Y. Zakharova, Sergey P. Medvedev, Sofia N. Pchelina, Suren M. Zakian

**Affiliations:** 1 Institute of Cytology and Genetics, Siberian Branch of Russian Academy of Sciences, Novosibirsk 630090, Russia; 2 Petersburg Nuclear Physics Institute named by B.P. Konstantinov of National Research Center «Kurchatov Institute», Gatchina 188300, Russia; 3 Department of Molecular Genetic and Nanobiological Technologies, Scientific and Research Centre, Pavlov First Saint-Petersburg State Medical University, Saint-Petersburg 197022, Russia; 4 Research Centre for Medical Genetics, Moscow 115522, Russia

**Keywords:** induced pluripotent stem cells, neural differentiation, dopaminergic neurons, glucocerebrosidase, mutation in the *GBA* gene, asymptomatic mutation carrier, ambroxol

## Abstract

*GBA* variants increase the risk of Parkinson’s disease (PD) by 10 times. The *GBA* gene encodes the lysosomal enzyme glucocerebrosidase (GCase). The p.N370S substitution causes a violation of the enzyme conformation, which affects its stability in the cell. We studied the biochemical characteristics of dopaminergic (DA) neurons generated from induced pluripotent stem cells (iPSCs) from a PD patient with the *GBA* p.N370S mutation (GBA-PD), an asymptomatic *GBA* p.N370S carrier (GBA-carrier), and two healthy donors (control). Using liquid chromatography with tandem mass spectrometry (LC-MS/MS), we measured the activity of six lysosomal enzymes (GCase, galactocerebrosidase (GALC), alpha-glucosidase (GAA), alpha-galactosidase (GLA), sphingomyelinase (ASM), and alpha-iduronidase (IDUA)) in iPSC-derived DA neurons from the GBA-PD and GBA-carrier. DA neurons from the *GBA* mutation carrier demonstrated decreased GCase activity compared to the control. The decrease was not associated with any changes in *GBA* expression levels in DA neurons. GCase activity was more markedly decreased in the DA neurons of GBA-PD patient compared to the GBA-carrier. The amount of GCase protein was decreased only in GBA-PD neurons. Additionally, alterations in the activity of the other lysosomal enzymes (GLA and IDUA) were found in GBA-PD neurons compared to GBA-carrier and control neurons. Further study of the molecular differences between the GBA-PD and the GBA-carrier is essential to investigate whether genetic factors or external conditions are the causes of the penetrance of the p.N370S *GBA* variant.

## 1. Introduction

Parkinson’s disease (PD) is the second most common neurodegenerative disease after Alzheimer’s disease. There are over 90 known genetic loci associated with the development of PD [1]. Among them, genetic variants in the *GBA* gene attract special attention due to their prevalence and the emerging risk of developing PD associated with a decrease in lysosomal function [2]. It is known that, in PD, the frequency of *GBA* mutations reaches 10%, while the risk of PD development among individuals carrying *GBA* mutations increases up to 20 times based on ethnicity [3,4]. However, the penetrance of PD among carriers of *GBA* pathogenic variants is estimated at 10–30%, indicating that the majority of mutation carriers will never develop PD. Therefore, it is extremely important to understand the pathological molecular mechanisms leading to the development of the disease to search for targets and possible approaches to the treatment of PD associated with mutations in the *GBA* gene (GBA-PD).

The *GBA* gene encodes the lysosomal enzyme beta-glucosylceramidase (GCase), which cleaves sphingolipids, in particular glucosylceramide, into glucose and ceramide. The homozygous state *GBA* mutations cause Gaucher disease, a systemic lysosomal storage disorder, characterized by a deficiency in GCase enzymatic activity and impaired glucolipids catabolism. More than 480 mutations are reported in the *GBA* gene (The Human Gene Mutation Database (HGMD) professional 2021.4.1, 5 March 2022). The two most common variants, N370S (43%) and L444P (20%), have different impacts on the GCase activity [5].

Both mutations are not located within the GCase active center but lead to GCase misfolding. Pharmacological chaperones are chemical compounds with low molecular weight, often hydrophobic, that can specifically bind to a mutant enzyme, correct its folding, and promote translocation into lysosomes [6]. Ambroxol, a well-known mucolytic agent, is currently one of the most promising GCase pharmacological chaperones. Ambroxol has been shown to restore GCase activity and protein levels, as well as reduce the lysosphingolipid concentration in different patient-derived cell types, including dopaminergic neurons [7,8,9,10]. Ambroxol has also demonstrated its efficiency in several ongoing clinical trials involving Gaucher disease and PD patients [11,12].

The mechanism of PD pathogenesis in *GBA* mutation carriers remains unclear. Previously, the decrease in GCase enzymatic activity in the blood of PD patients with mutations in the *GBA* gene was reported [13,14,15]. Recently, we have shown that GCase activity in blood is decreased in *GBA* mutation carriers, independently of PD status [16].

The technology of reprogramming somatic cells, such as peripheral blood mononuclear cells (PBMCs), to a pluripotent state makes it possible to create bioresource collections of immortal patient-specific induced pluripotent stem cells (iPSCs) with various pathologies caused by genetic mutations. The advantage of the technology is its capability to produce any type of highly specialized cell through the direct differentiation of iPSCs. This method allows for the in vitro modeling of almost any genetic disorder by generating relevant cell types suffering from the disease of interest. Today, numerous iPSCs-derived dopamine neuronal cells have been obtained from patients with Gaucher disease and GBA-PD [17,18,19,20]. The N370S mutation lines are characterized by reduced GCase activity and protein levels compared to controls [19,20]. Recently, the decrease in GCase activity was also demonstrated in a cholinergic N370S *GBA* mutation model [7]. However, there is only one study that estimated the GCase activity in a neuronal model of healthy *GBA* mutation carriers [21].

Here, we obtained dopaminergic (DA) neurons from iPSCs from GBA-PD and non-manifesting GBA-carrier individuals to compare the biochemical effects of the N370S *GBA* mutation and evaluate the influence of ambroxol on *GBA* expression, GCase activity, and protein levels. We measured GCase enzymatic activity as well as the activity of five other lysosomal hydrolases (galactocerebrosidase (GALC, EC 3.2.1.46, deficient in Krabbe disease), alpha-glucosidase (GAA, EC 3.2.1.20, deficient in Pompe disease), alpha-galactosidase (GLA, EC 3.2.1.22, deficient in Fabry disease), sphingomyelinase (ASM, EC 3.1.4.12, deficient in Niemann–Pick disease types A and B), and alpha-iduronidase (IDUA, EC 3.2.1.76, deficient in mucopolysaccharidosis type I)) in DA neurons from the GBA-PD and GBA-carrier. Further studies of the molecular differences between the GBA-PD and GBA-carrier DA neurons are essential for an investigation into whether genetic factors or external conditions play a role in the penetrance of the p.N370S *GBA* variant.

## 2. Results

The analysis of the clinical exome sequencing data of two individuals (a 56-year-old woman (PD30) with PD and her healthy 32-year-old son (PD31)) was performed. Bioinformatic analysis revealed that both patients harbor a pathogenic heterozygous missense mutation *c.1226A>G* (p.N370S, rs76763715) in the *GBA* gene. Here, we referred to the PD30 patient as a GBA-PD and the asymptomatic carrier PD31 as a GBA-carrier.

We identified around 15000 SNVs in each patient, including around 8500 SNVs reported in the ClinVar database (Table S2). The clinically significant SNVs are reported in Table S3. The pathogenic and risk factor genetic variants in the *SOX2*, *FBXO38,* and *RNASEL* genes are the possible candidates for the modifiers of the disease manifestation, since the mentioned SNVs were found in the GBA-PD patient’s exome and were absent in the GBA-carrier (*FBXO38* and *RNASEL*), or were present in the homozygous state of GBA-PD and are heterozygous in the GBA-carrier (*SOX2*) (Table S3). The search for polymorphisms in PD-associated genes revealed 48 SNVs, all of which are marked as benign in the ClinVar database (Table S4). Therefore, it seems unlikely that the identified SNVs in PD-associated genes have an effect on the *GBA* mutation penetrance.

PBMCs-derived IPSC lines from the GBA-PD patient (https://hpscreg.eu/cell-line/ICGi034-A; https://hpscreg.eu/cell-line/ICGi034-B; https://hpscreg.eu/cell-line/ICGi034-C; all accessed on 17 February 2023) [22] and GBA-carrier (https://hpscreg.eu/cell-line/ICGi039-A; https://hpscreg.eu/cell-line/ICGi039-B; https://hpscreg.eu/cell-line/ICGi039-C; all accessed on 17 February 2023) were characterized and registered in the Human Pluripotent Stem Cell Registry (hPSCreg). The detailed characteristics of three GBA-carrier lines are presented in the Supplementary Materials (Figures S1–S3). As the control lines, we used iPSCs obtained from healthy individuals (https://hpscreg.eu/cell-line/ICGi021-A; https://hpscreg.eu/cell-line/ICGi022-A; all accessed on 17 February 2023) [23].

To create a cell platform for drug screening, the cells of iPSC lines derived from GBA-PD, GBA-carrier, and control lines were differentiated into DA neurons, which are the relevant type of cells affected in PD.

### 2.1. Directed Differentiation of iPSCs to DA Neurons and Characteristics of the Obtained Neurons

**Figure 1:**
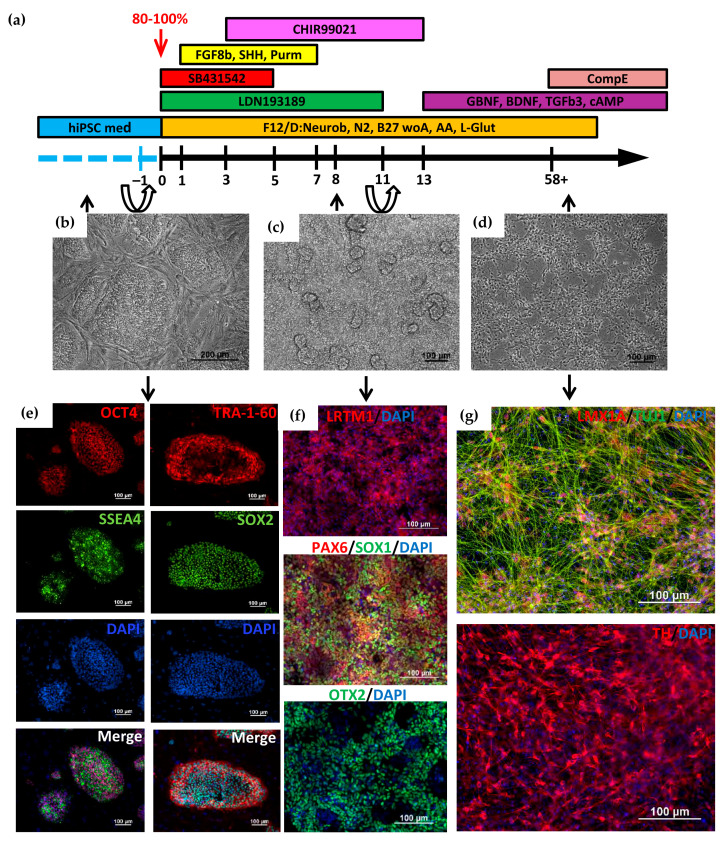
Directed differentiation of induced pluripotent stem cells (iPSCs) into dopaminergic (DA) neurons. (**a**) Scheme of iPSCs’ differentiation into DA neurons. Cell passaging was performed on –1st and 11th days of differentiation; (**b**) Morphology of iPSCs cultured on MEF; (**c**) Monolayer of neuroectodermal cells on day 8 of differentiation; (**d**) Morphology of terminally differentiated neurons. Phase contrast (**b**–**d**); (**e**) Immunofluorescent staining of iPSCs for pluripotency markers: transcription factors OCT4 (red signal), SOX1 (green signal) and surface markers TRA-1-60 (red signal), SSEA4 (green signal); (**f**) Immunofluorescence staining for DA neuron progenitor marker LRTM1 (red signal) and early neuroectoderm markers: PAX6 (red signal), OTX2 (green signal), SOX1 (green signal); (**g**) Immunofluorescent staining for markers of terminally differentiated DA neurons: tyrosine hydroxylase (TH, red signal) and LMX1A (red signal). Nuclei are stained with DAPI (blue signal). Scale bar: 100 µm.

Directed differentiation was carried out according to the previously published protocols [24,25,26,27,28]. The cells were cultured in a dense monolayer on the growth factor-reduced extracellular matrix Matrigel (Matrigel-GFR) in the culture medium containing growth factors and inhibitors that promote the triggering of signaling pathways that simulate neurulation during the early development of the embryo in vivo. Figure 1a shows the complete differentiation scheme. At the preparatory stage, iPSCs (Figure 1b,e) were transferred from mouse embryonic fibroblasts (MEF) onto Matrigel-GFR. It should be noted that the most efficient differentiation occurs when a dense monolayer of iPSCs (90–100% of confluency) is plated onto Matrigel for 24 h before the start of differentiation. At the first stage, iPSCs differentiation in the neuroectodermal direction was stimulated using double SMAD inhibition [25], with the addition of the small molecules LDN193189 and SB431542. These factors suppress pluripotency gene expression and inhibit differentiation in the mesodermal and endodermal directions. The addition of CHIR99021 on days 3–13 of differentiation directs neural progenitor cells to midbrain cells and contributes to a better output of DA neurons. Sonic hedgehog (SHH), FGF8b, and purmorphamine also increase the efficiency of directed differentiation into DA neurons [28,29,30]. During the 11 days of cultivation, there was a multifold increase in cell number with the formation of convex structures consisting of one type of cell (Figure 1c). These cells express the early markers of neuroectoderm (PAX6, SOX1, and OTX2), as well as the marker of DA neuron precursors, LRTM1 [31] (Figure 1f).

On day 11, a dense monolayer of cells was disaggregated and seeded onto Matrigel-GFR with the addition of a ROCK inhibitor to the culture medium. On the 13th day of differentiation, the factors necessary for obtaining mature neurons (BDNF, TGFb3, GDNF, and dbcAMP) were added to the cells, and cell cultivation continued at a high density with weekly passaging and the freezing of some leftover cells.

The advantage of this protocol is the possibility of collecting a large mass of DA neuron progenitor cells, some of which can be frozen and, if necessary, thawed, and further terminal differentiation can be carried out. This greatly simplifies further experiments on obtaining terminally differentiated DA neurons and their use in various experiments.

**Figure 2:**
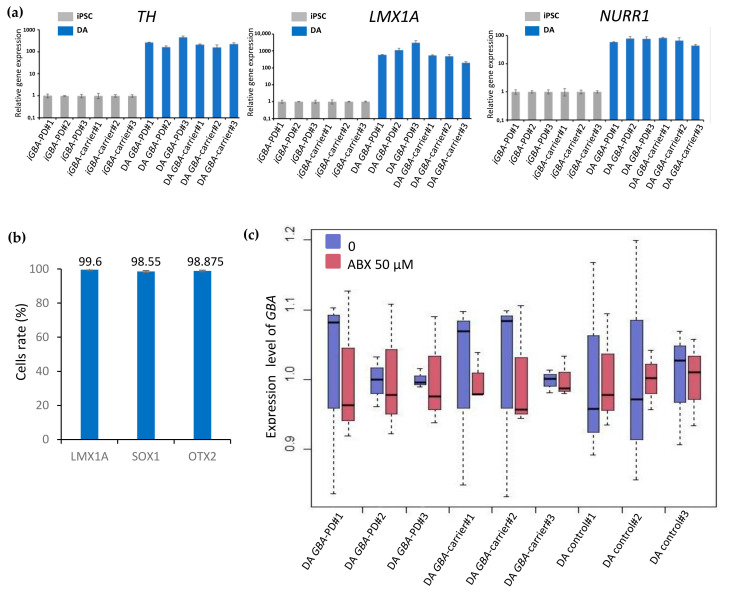
Characterization of DA neurons and their precursors. (**a**) qPCR gene expression analysis of DA neuron markers (TH, LMX1A, and NURR1) in neurons at days 50–60 of differentiation (blue) compared to iPSCs (gray) (*n* = 3); (**b**) flow cytometry analysis of the DA neuron precursor cells at day 34 of differentiation for early marker (LMX1A) and specific neuroectodermal markers (SOX1 and OTX2), *n* = 4; (**c**) expression level of *GBA* in DA neurons obtained from two *GBA* mutation carriers (GBA-PD and GBA-carrier) and “healthy” controls before (blue) and after (red) ambroxol (ABX) treatment.

We analyzed the expression of the genes specific for neuronal DA precursors (LMX1A) and neuroectodermal markers (SOX1 and OTX2) in progenitor cells at differentiation day 34 after 23 days of cultivation at high density in the presence of BDNF, GDNF, TGFb3, and cAMP. Using flow cytometry, we showed that the majority of differentiated cells (98.5–99.6%) were precursors of DA neurons (Figure 2b and Figure S4).

For terminal differentiation into DA neurons, neural progenitors were seeded in low density with the addition of the gamma-secretase inhibitor Compound E to the culture medium. Neurons were characterized by immunofluorescence staining and qPCR, which demonstrated expression of tyrosine hydroxylase (TH), a specific marker of DA neurons, and DA-specific transcription factors LMX1A, LRTM1 [28], and a member of the nuclear receptor superfamily of transcription factors NURR1 [29], as well as the common neuronal marker Tubulin β3 (TUBB3/TUJ1) (Figure 1g). The qPCR analysis showed a significant increase in the expression of the *TH*, *LMX1A,* and *NURR1* genes in DA neurons compared to the expression in iPSCs (Figure 2a).

### 2.2. Analysis of the GBA Gene Expression in DA Neurons before and after Treatment with Ambroxol

The resulting neural derivatives were cultured for 3 weeks in the presence of 50 μM ambroxol. We performed qPCR analysis of *GBA* expression in DA neurons derived from the GBA-PD, GBA-carrier, and control iPSCs before and after ambroxol treatment, in triplicate for each group. *B2M* and *TFRc* served as reference genes for data normalization. Our data demonstrated the absence of significant differences in *GBA* expression between the studied groups (Figure 2c).

### 2.3. Lysosomal Enzymes Activity in DA Neurons

**Figure 3:**
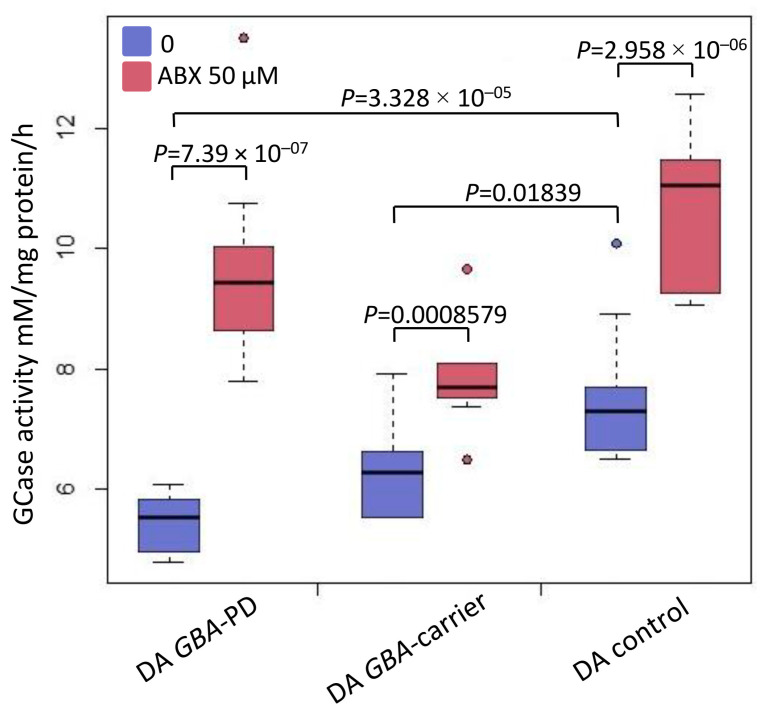
Evaluation of the ambroxol effect on the restoration of GCase activity. GCase activity assessed by liquid chromatography coupled with tandem mass spectrometry (LC-MS/MS) in iPSC-derived DA neurons from GBA-PD and GBA-carrier and “healthy” controls before (blue) and after (red) ambroxol (ABX) treatment, *p*-value < 0.05, *n* = 12. All experiments were performed on three iPSC lines for each patient.

Measuring the enzymatic activities of six lysosomal enzymes (GCase, GALC, GAA, GLA, ASM, and IDUA) was carried out by liquid chromatography combined with tandem mass spectrometry (LC-MS/MS). We used substrates and internal standards of the Centers for Disease Control and Prevention (Atlanta, GA, USA) as described earlier for primary macrophage culture [15] with modifications. GCase activity was lower in iPSCs-derived DA neurons from the GBA-PD and GBA-carrier compared to controls (*p*-value < 0.0001 and *p*-value = 0.018, respectively) (Table 1) (Figure 3, blue). Moreover, we found differences in GCase activity between the GBA-PD and GBA-carrier (*p*-value = 0.025) (Table 1). In our study, activity of the GLA and IDUA enzymes was higher in DA neurons from GBA-PD patients compared to controls (*p*-value = 0.008 and *p*-value < 0.0001, respectively) as well as the GBA-carrier (*p*-value = 0.002 and *p*-value < 0.0001, respectively) (Table 1). GALC activity was lower in DA neurons from the GBA-PD compared to the GBA-carrier (*p*-value = 0.045) (Table 1).

### 2.4. Analysis of GCase Activity in DA Neurons before and after Treatment with Ambroxol

We analyzed GCase enzyme activity before and after ambroxol treatment. Ambroxol caused a significant increase in GCase activity in iPSCs-derived DA neurons of both mutation carriers (GBA-PD and GBA-carrier) compared to untreated cells (Figure 3, red).

### 2.5. Western Blot Analysis before and after Treatment with Ambroxol 

**Figure 4:**
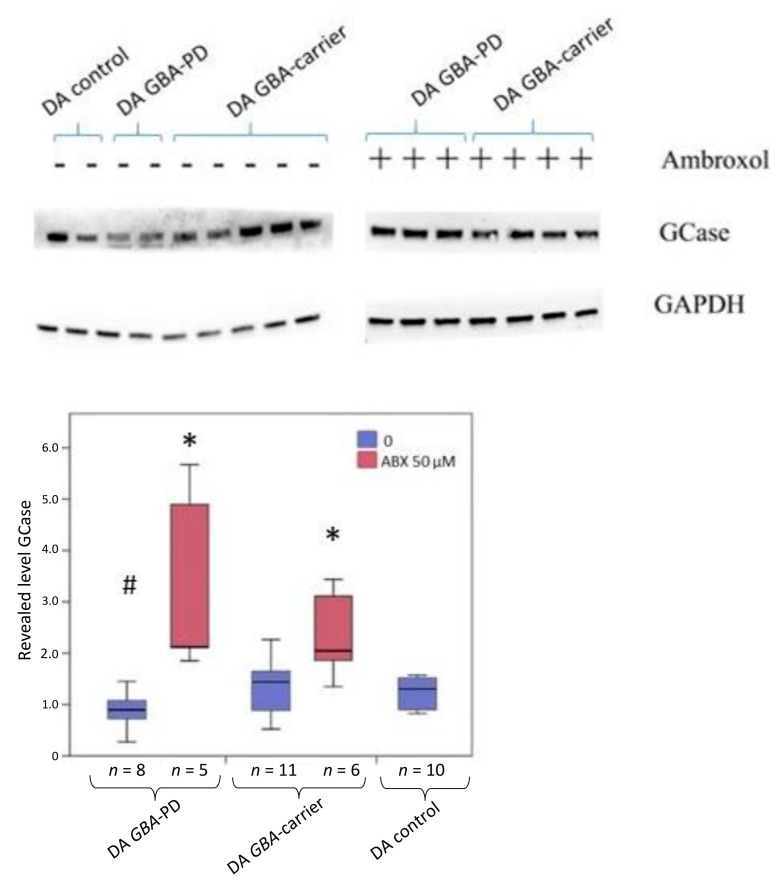
Evaluation of the ambroxol effect on the restoration of GCase protein level. Western blot analysis of GCase level and quantification of the results as the ratio of GCase to GAPDH in iPSC-derived DA neurons. Statistically significant differences between the untreated and treated cells: * *p* < 0.05 compared to untreated cells, # *p* < 0.05 compared to control. ABX—ambroxol. All experiments were performed on three iPSC lines for each patient.

Western blot analysis was performed on samples of iPSCs-derived DA neurons with the *GBA* mutation and the control group to quantify the GCase protein (Figure 4 and Figure S5). We showed a decrease in the GCase protein level in neurons obtained from GBA-PD compared to the control (*p*-value = 0.021) (Figure 4, blue). Ambroxol led to a significant increase in the amount of the protein, both in the DA neurons of the GBA-carrier (*p*-value = 0.038) and the GBA-PD (*p*-value = 0.021) (Figure 4, red).

## 3. Discussion

Our study describes the difference in biochemical characteristics between DA neurons obtained by the differentiation of iPSCs from heterozygous *GBA* N370S carriers with and without PD and the effect of ambroxol on GCase activity and protein level.

PBMC reprogramming technology allowed us to generate iPSC lines from two patients carrying the heterozygous N370S mutation in the *GBA* gene (https://hpscreg.eu/cell-line/ICGi034-A; https://hpscreg.eu/cell-line/ICGi034-B; https://hpscreg.eu/cell-line/ICGi034-C and https://hpscreg.eu/cell-line/ICGi039-A; https://hpscreg.eu/cell-line/ICGi039-B; https://hpscreg.eu/cell-line/ICGi039-C; all accessed on 17 February 2023). The iPSC lines obtained meet all the criteria for pluripotent stem cells. These lines, as well as previously obtained control iPSCs from healthy individuals, were differentiated into DA neurons using growth factors and inhibitors that simulate events occurring in the embryo during neurogenesis in vivo. Expression of the transcription factors, such as PAX6, SOX1, and OTX2, at the early stages of differentiation indicated that iPSCs were successfully differentiated in the neuroectodermal direction. Moreover, we carried out the long-term cultivation of cells at high density for three weeks in the presence of BDNF, GDNF, TGFb3, and cAMP. It was shown that 98.5–99.6% of cells were SOX1-, OTX2-, and LMX1A-positive, which indicates that the cells can be maintained in the state of precursors for a long time. Cultivation in a low density in the presence of Compound E caused maturation and aging of neurons, resulting in a significant increase in the expression of specific markers of DA neurons *TH*, *NURR1* and *LMX1A*.

Previously, lower GCase activity in GBA-PD compared to controls was shown using various biological samples and in vitro models (including blood, CSF, postmortem brain, mononuclear cells, fibroblasts, and iPSC-derived neurons) [10,17,32,33,34,35]. Later, we and other researchers assessed GCase activity in the blood and mononuclear cells of GBA-carriers and demonstrated that GCase activity is also reduced in healthy *GBA* mutation carriers without PD and could not discriminate PD status in *GBA* mutation carriers [16,36]. The lysosphingolipid concentration was suggested as a more sensitive biomarker of GCase deficiency in patients with Gaucher disease [37]. Few studies compared lysosphingolipid concentrations in the blood of GBA-PD and GBA-carriers [16,38]. We were the first to demonstrate that GBA-PD patients had increased blood hexosylsphingosine concentrations compared to GBA-carriers [16]. However, biochemical characteristics in the blood may not reflect those in DA neurons.

Here, for the first time, we measured the activities of six lysosomal enzymes (GCase, GALC, GAA, GLA, ASM, and IDUA) in DA neurons from the GBA-PD and GBA-carrier. In this study, we found that GBA-PD had lower GCase activity and higher GLA and IDUA activities compared to controls as well as the GBA-carrier. Recent studies in sporadic PD have identified variants in multiple genes linked to lysosomal storage disorders [39]. The activity of GLA (deficient in Fabry disease) in the blood was found to be reduced in PD [40,41,42] and GBA-PD patients [15]. Interestingly, elevated GLA activity in the blood was reported in LRRK2-PD patients [41] and patients with one of the forms of synucleinopathies (multiple system atrophy) [43]. Previously, we showed increased GLA expression levels and activity in the blood of patients with multiple system atrophy but not in PD [43]. Mutations in the ASM gene (*SMPD1*), responsible for Niemann-Pick type A/B, have also been associated with PD [44]. Previously, Alcalay et al. showed decreased ASM activity and increased alpha-synuclein levels in the blood of PD patients [44]. In our study, we did not observe any differences in ASM and GALC activity in *GBA* mutation carriers (with and without PD). The reason for the increased GLA and IDUA activity in DA neurons in GBA-PD patients is currently unclear. Further research is required to assess the role of lysosomal enzymes and their interactions in the pathogenesis of GBA-PD.

We discovered decreased GCase activity in DA neurons of N370S *GBA* mutation carriers independent of PD status. However, a reduction in GCase activity was more evident in DA neurons obtained from PD patients than in the non-manifesting GBA-carrier. Woodard et al. studied midbrain dopaminergic neurons derived from twins carrying heterozygous *GBA* N370S and showed lower GCase activity and protein levels as well as an increase in alpha-synuclein levels in GBA-PDs and GBA-carriers [21]. Compared to Woodard et al.’s observations, in the present study, we showed reduced GCase protein levels in DA neurons from the GBA-PD compared to controls, but not in DA neurons from the healthy GBA-carrier. Previously, McNeill et al. showed reduced GCase activity and protein level in fibroblasts from *GBA* mutation carriers (with and without PD) [10]. In contrast to our data, McNeill did not report any differences between GBA-PD and GBA-carrier iPSC-derived DA neurons in GCase activity. It should be noted that in McNeill’s study, both groups included subjects with different severities of *GBA* mutations. On the other hand, we could not exclude the possibility that a reciprocal relationship between alpha-synuclein level and GCase activity resulting in a GCase trafficking defect, lysosomal dysfunction, and substrate accumulation depends on cell type and could be detected in DA neurons. While we have not measured the lysoshingolipid and alpha-synuclein levels, further investigations are warranted to clarify the relationship between GCase deficiency and neurodegeneration in PD.

A promising GCase-targeted therapy is molecular chaperones. Two types of molecular chaperones exist: inhibitory chaperones, which bind to the active site of the GCase protein, and noninhibitory chaperones, which bind to an alternate site on the GCase surface [45]. Ambroxol is a pH-dependent mix-type chaperone of GCase [46]. Previously, we constructed an atomistic model of the mutant N370S GCase, and using molecular docking and molecular dynamics, we confirmed that ambroxol appeared to be a mixed-type inhibitor of GCase. A novel allosteric binding site for ambroxol on the GCase surface was identified, located next to the substituent residue S370 under loop L1 (311–319 amino acid residues) [9]. Previously, ambroxol was shown not only to increase GCase activity but also to reduce lysosphingolipids and alpha-synuclein pathology in DA and cholinergic neurons of N370S GBA-PD [20,46]. The ambroxol efficiency was also demonstrated in patent-derived fibroblasts [10] and primary macrophages culture [9] as well as on animal models of GBA-PD (*Drosophila*, mouse midbrain and non-human primate brain) [47,48,49]. In accordance with all previous data, we showed that ambroxol successfully chaperoned GCase activity in iPSC-derived DA neurons from GBA-PD patient. In addition, we showed the same effect on iPSC-derived DA neurons from healthy *GBA* mutation carrier. Ambroxol increased both GCase activity and protein level in DA neurons independently from PD status and did not alter *GBA* expression.

Thus, we first showed that biochemical characteristics, namely the activities of lysosomal hydrolases, may differ in iPSC-derived DA neurons from GBA-PD patients and healthy GBA-carriers.

## 4. Materials and Methods

### 4.1. Ethics

The biomaterial was collected at the FSBI Federal Neurosurgical Center with the permission of the ethics committee (Protocol number 1, 14 March 2017) and after the patients signed the informed consent and information sheet. All research is conducted anonymously.

### 4.2. Whole-Exome Sequencing and Genotyping

Clinical exome sequencing was performed on DNA samples from the patients’ PBMCs. The library was made using the NEBNext Ultra DNA Library Prep Kit (New England Biolabs, Ipswich, MA, USA) for Illumina, followed by double barcoding with NEBNext Multiplex Oligos for Illumina (New England Biolabs, Ipswich, MA, USA), quality control with the Agilent Bioanalyzer 2100, enrichment with the SureSelectXT Target Enrichment System (Agilent Technologies, Santa Clara, CA, USA), and sequencing in Rapid Run Mode. Raw reads obtained from Illumina HiSeq 2500 (PRJNA563295, BioSample accession SAMN22788974 (https://www.ncbi.nlm.nih.gov/biosample/22788974, accessed on 17 February 2023), and SAMN26587088 (https://www.ncbi.nlm.nih.gov/biosample/26587088, accessed on 17 February 2023)) were cleaned, filtered, and aligned to the GRCh37 human reference genome with appropriate quality control. Germline variants were called with the GATK pipeline using best practices, annotated with several databases, carefully filtered, and prioritized. The Genomenal NGSWizard software (https://genomenal.com/, accessed on 17 February 2023) was used to run data processing pipelines. Pipelines are the launch of the following programs: FastX Toolkit for reads processing, BWA MEM for aligning reads to the reference genome (hg38), GATK v.4.1.0.0 for calling SNP with subsequent annotation.

The single nucleotide polymorphism rs76763715 *(c.1226A>G*, p.N370S), which was found in the *GBA* gene, was confirmed by Sanger sequencing. PCR was performed using primers from Table S1. Reactions were run on a T100 Thermal Cycler (Bio-Rad Laboratories, Singapore) using BioMaster HS-Taq PCR-Color (2×) (Biolabmix, Novosibirsk, Russia) with the following program: 95 °C for 3 min; 35 cycles: 95 °C for 30 s; 60 °C for 30 s; 72 °C for 30 s; and 72 °C for 5 min. Sanger sequencing reactions were performed with the Big Dye Terminator V. 3.1. Cycle Sequencing Kit (Applied Biosystems, Austin, TX, USA) and analyzed on an ABI 3130XL Genetic Analyzer at the SB RAS Genomics Core Facility (http://www.niboch.nsc.ru/doku.php/corefacility, accessed on 17 February 2023).

### 4.3. DNA Isolation

DNA was isolated using Quick-DNA Miniprep Kit (Zymo Research, Irvine, CA, USA) for STR analysis or extracted by QuickExtract™ DNA Extraction Solution (Lucigen, Madison, WI, USA) for the *GBA* gene mutation analysis PCR, episome and mycoplasma detection.

### 4.4. Cultivation of iPSCs

iPSCs were cultivated in the growth medium containing KnockOut DMEM, 15% KnockOut Serum Replacement, GlutaMAX-I, 0.1 mM NEAA, 1% penicillin-streptomycin (all from Thermo Fisher Scientific, Waltham, MA, USA), 0.1 mM 2-mce (Sigma-Aldrich, Darmstadt, Germany), and 10 ng/mL bFGF (SCI Store, Moscow, Russia) onto a gelatin-coated plate with mouse embryonic fibroblasts (MEF) treated with Mitomycin C from Streptomyces caespitosus (Sigma-Aldrich, Darmstadt, Germany). For DA differentiation, iPSCs were passaged onto Matrigel-GFR (Corning, New York, NY, USA) in Essential 8 Medium (Thermo Fisher Scientific, Waltham, MA, USA). iPSCs were passaged 2 times a week at a ratio of 1:8–1:10 with the addition of 2 μg/mL Thiazovivin (Sigma-Aldrich, Darmstadt, Germany). Dissociation of iPSC colonies was carried out using TrypLE (Thermo Fisher Scientific, Waltham, MA, USA). Cells were cultured in a CO_2_ incubator (37 °C, 5% CO_2_).

### 4.5. Differentiation of Patient-Specific iPSCs into DA Neurons

Directed differentiation of iPSCs into DA neurons was performed according to a previously published protocol [26], with modifications. The iPSCs were plated at a high density onto Matrigel-GFR-treated plates so that the cell monolayer reached 80–100% confluency the next day. The culture medium was changed to one containing F12/DMEM:Neurobasal (1:1), 0.5× N-2 Supplement, 0.5× B-27 Supplement minus vitamin A, GlutaMAX™ Supplement, 1× penicillin-streptomycin (all from Thermo Fisher Scientific, Waltham, MA, USA), and 200 µM ascorbic acid (Sigma-Aldrich, Darmstadt, Germany). Next, growth factors, inhibitors, and small molecules were added to the cells according to the scheme (Figure 1). Concentrations of growth factors, inhibitors, and small molecules are presented in Table 2. On the 11th day of differentiation, the cell monolayers were passaged using StemPro™ Accutase™ (Thermo Fisher Scientific, Waltham, MA, USA) in the ratio 1:2 with the addition of ROCK inhibitor. On the next day, BDNF, GDNF, TGFb3, and dbcAMP (all from PeproTech, Cranbury, NJ, USA) were added to the culture medium. Cells were passaged once a week in the ratio of 1:3–1:4. After 30 days, the cells were seeded at a density of 10^5^ cells/cm^2^ in the medium containing 0.1 µM Compound E (Sigma-Aldrich, Darmstadt, Germany) for 10–14 days.

### 4.6. Immunofluorescent Analysis

Immunofluorescent staining was performed according to the previously described procedure [50]. Briefly, cells were grown on cell culture imaging plates for microscopy, fixed for 10 min in 4% PFA, and washed twice in DPBS. Permeabilization was performed in 0.5% Triton ×100 for 30 min, and nonspecificity was blocked with 1% BSA for 30 min. Exposure with primary antibodies diluted in 1% BSA was performed overnight at +4 °C. The secondary antibodies were then exposed for 1.5 h at room temperature. The list of antibodies is presented in Table 3.

### 4.7. Flow Cytometry Analysis 

Cells were disaggregated with TrypLE Express and fixed in 2% paraformaldehyde (Sigma-Aldrich, Darmstadt, Germany). The cell membrane was permeabilized in 100% methanol at −20 °C for 10 min, washed once with DPBS (Biolot, Saint-Petersburg, Russia), and stained with the first antibodies diluted in 1% BSA in DPBS at 4 °C overnight. The next day, cells were washed once with DPBS, and secondary antibodies diluted in 1% BSA in DPBS were added for 1 h. Secondary antibodies were used as isotype controls. A cell count was performed by the BD FACSAria™ III (BD Biosciences, Franklin Lakes, NJ, USA) using the BD FACSDiva software. All the measurements were made using four replicates. The list of antibodies used in the FACS analysis is presented in Table 3.

### 4.8. Quantitative RT-PCR

Reverse transcription of 1 µg of RNA was performed using M-MuLV revertase (Biolabmix, Novosibirsk, Russia). Quantitative PCR was performed on a LightCycler 480 real-time PCR system (Roche, Basel, Switzerland) with a BioMaster HS-qPCR SYBR Blue 2× kit (Biolabmix, Novosibirsk, Russia) using the following program: 95 °C for 5 min; 40 cycles at 95 °C for 10 s and 60 °C for 1 min. *ACTB*, *B2M*, and *TFRC* were chosen as reference genes. Quantitative analysis of the qPCR results was performed using the generalized ΔΔCt method, taking into account the reaction efficiency calculated from the results of constructing a five-point calibration curve [51]. The list of primers is presented in Table 4.

### 4.9. Ambroxol Treatment of DA Neurons

To examine the effects of ambroxol treatment, DA neurons derived from patient-specific iPSCs were grown in a complete medium containing 50 µM ambroxol (Sigma-Aldrich, Darmstadt, Germany) for 21 days. Each line of DA neurons was cultured in 3 wells of a 12-well plate with ambroxol and 3 wells without ambroxol (null point) with a daily change of the medium.

### 4.10. Western Blot Analysis

Cells were lysed with a total protein extraction kit (Millipore, Burlington, VT, USA). Total protein concentration was measured using a Pierce BSA Protein Assay kit (Thermo Scientific, Waltham, MA, USA). A total of 30 µg of total protein extract was loaded onto a 7.5% mini-protean TGX stain-free precast gel (Bio-Rad Laboratories, Hercules, CA, USA) with Tris/Glycine/SDS running buffer (Bio-Rad Laboratories, Hercules, USA) and transferred to a PVDF membrane (Bio-Rad Laboratories, Hercules, CA, USA). The membrane was probed with primary antibodies (rabbit anti-GBA monoclonal antibody (1:500, ab125065, Abcam, Cambridge, UK) or rabbit anti-glyceraldehyde 3-phosphate dehydrogenase (GAPDH) polyclonal antibody (1:18,000, SAB2108266, Sigma-Aldrich, Darmstadt, Germany). The goat anti-rabbit HRP conjugate (1:5000, ab6721, Abcam, Cambridge, UK) was used to detect both anti-GBA and anti-GAPDH antibodies. Digital images were obtained using the chemiluminescence system ChemiDoc (Bio-Rad Laboratories, Hercules, CA, USA) and quantified using ImageJ software.

### 4.11. Quantification of Lysosomal Enzymes Activity in DA-Neurons

The activity of six lysosomal enzymes—GCase, GALC, GAA, GLA, ASM, and IDUA—was measured in triplicate for each line of DA neurons derived from patient-specific iPSC using liquid chromatography coupled with tandem mass spectrometry (LC-MS/MS) as described previously [9,53]. The neurons were lyophilized and then resuspended in DPBS. Enzyme activities were normalized to the total protein concentration.

### 4.12. Statistical Analysis

Statistical significance in value differences among experimental groups was calculated by an ANOVA test in Microsoft Excel (Microsoft Office 2013) software and a Wilcoxon’s *t*-test in R version 4.2.2 (R Core Team, 2021, Vienna, Austria).

## 5. Conclusions

The 10–30% penetrance of *GBA* genetic variants raises the question of why some carriers get sick and others do not. Asymptomatic *GBA* mutation carriers are an interesting group for genetic studies, as they might carry protective genetic variants, either generally neuroprotective or counteracting the effect of lower GCase activity. We have shown a decrease in GCase activity in iPSC-derived DA neurons in both asymptomatic N370S *GBA* carriers and PD patients. A decrease in the amount of GCase protein was shown in GBA-PD neurons but not in GBA-carrier. Moreover, GBA-PD DA neurons are characterized by a disbalance in other lysosomal enzyme activities and have higher GLA and IDUA activities compared to both control and GBA-carrier DA neurons. The imbalance of sphingolipid metabolism carried out by lysosomal enzymes may lead to lysosomal dysfunction, which could promote the pathogenesis of diseases associated with GCase deficiency.

## Figures and Tables

**Table 1:** Activities of the lysosomal enzymes are presented as medians (min–max).

Lysosomal Enzyme	GBA-PD (N370S/WT)	GBA-Carrier (N370S/WT)	Controls
GCase, nM/mg of protein/h	5.8 (4.8–6.9) * *p* < 0.0001 # *p* = 0.025	6.6 (5.5–7.9) * *p* = 0.018	7.6 (6.5–10.1)
GALC, nM/mg of protein/h	8.5 (7.3–10.6) # *p* = 0.045	10.6 (6.4–16.3)	8.4 (6.6–13.1)
GAA, nM/mg of protein/h	17.2 (12.4–20.3)	17.6 (14.8–19.3)	16.5 (8.8–18.6)
GLA, nM/mg of protein/h	12.2 (8.9–14.6) * *p* = 0.008 # *p* = 0.002	9.6 (7.6–10.5)	9.8 (8.8–12.1)
ASM, nM/mg of protein/h	9.5 (6.9–11.1)	9.9 (8.8–11.9)	8.9 (7.8–11.8)
IDUA, nM/mg of protein/h	20.9 (18.2–23.1) * *p* < 0.0001 # *p* < 0.0001	12.1 (8.4–16.9)	10.9 (6.9–20.9)

* Compared to controls, # Compared to GBA-carrier.

**Table 2:** List of used growth factors, inhibitors and small molecules for DA-neuron differentiation.

Substance	Company	Concentration	Days
LDN193189	Sigma-Aldrich, Darmstadt, Germany	100 nM	0–11
SB431542	Abcam, Cambridge, UK	10 µM	0–5
Purmorphamine	Tocris, Ellisville, MO, USA	2 µM	1–7
SHH C25II	PeproTech, Cranbury, NJ, USA	100 ng/mL	1–7
FGF8b	PeproTech, Cranbury, NJ, USA	100 ng/mL	1–7
CHIR99021	Sigma-Aldrich, Darmstadt, Germany	3 µM	3–13
BDNF	PeproTech, Cranbury, NJ, USA	20 ng/mL	13–to …
GDNF	PeproTech, Cranbury, NJ, USA	20 ng/mL	13–to …
TGFb3	PeproTech, Cranbury, NJ, USA	1 ng/mL	13–to …
dbcAMP	PeproTech, Cranbury, NJ, USA	0.5 mM	13–to …
Compound E	Millipore, Burlington, VT, USA	0.1 µM	terminal stage

**Table 3:** List of used antibodies.

Antibodies	Company	Cat. Ref.	Raised/Type	Dilution
Primary antibodies				
Anti-OCT4	Abcam, Cambridge, UK	ab18976	IgG rabbit polyclonal	1:200
Anti-SOX2	Cell Signaling, Danvers, MA, USA	3579	IgG rabbit polyclonal	1:500
Anti-SSEA4	Abcam, Cambridge, UK	ab16287	IgG3 mouse monoclonal	1:200
Anti-TRA-1-60	Abcam, Cambridge, UK	ab16288	IgM mouse monoclonal	1:200
Anti-LRTM1	EpiGentek, Farmingdale, NY, USA	A67852	IgG rabbit polyclonal	1:100
Anti-PAX6	Santa Cruz Biotechnology, Dallas, TX, USA	sc-81649	IgM mouse monoclonal	1:50
Anti-SOX1	R&D systems, Minneapolis, MN, USA	AF3369	IgG goat polyclonal	1:200
Anti-OTX2	R&D systems, Minneapolis, MN, USA	AF1979	IgG goat polyclonal	1:400
Anti-TH	Millipore, Burlington, VT, USA	AB152	IgG rabbit polyclonal	1:400
Anti-LMX1A	Abcam, Cambridge, UK	ab139726	IgG rabbit polyclonal	1:100
Anti-TUJ1	Covance, Princeton, NJ, USA	MMS-435P	IgG2a mouse monoclonal	1:1000
Secondary antibodies				
Alexa Fluor 488 goat anti-rabbit IgG (H+L)	Thermo Fisher Scientific, Waltham, MA, USA	A11008	Made in goat	1:400
Alexa Fluor 568 goat anti-rabbit IgG (H+L)	Thermo Fisher Scientific, Waltham, MA, USA	A11011	Made in goat	1:400
Alexa Fluor 488 goat anti-mouse IgG (H+L)	Thermo Fisher Scientific, Waltham, MA, USA	A11029	Made in goat	1:400
Alexa Fluor 568 goat anti-mouse IgG (H+L)	Thermo Fisher Scientific, Waltham, MA, USA	A11031	Made in goat	1:400
Alexa Fluor 488 goat anti-mouse IgG2a	Thermo Fisher Scientific, Waltham, MA, USA	A21131	Made in goat	1:400
Alexa Fluor 488 donkey anti-goat IgG (H+L)	Thermo Fisher Scientific, Waltham, MA, USA	A11055	Made in donkey	1:400
Alexa Fluor 594 rabbit anti-mouse IgG (H+L)	Thermo Fisher Scientific, Waltham, MA, USA	A11062	Made in rabbit	1:400

**Table 4:** List of used primers.

	Target	Forward/Reverse Primer (5′-3′)
House-keeping gene (RT-qPCR)	*beta-2-microglobulin*	TAGCTGTGCTCGCGCTACT/ TCTCTGCTGGATGACGTGAG
	*TFRC*	AGCAGTTGGCTGTTGTACCTCTC/ GTCGCTGGTCAGTTCGTGATT
	*ACTB*	GTGCGTGACATTAAGGAGAAG/ GAAGGAAGGCTGGAAGAGTG
Gene expression analysis (RT-qPCR) [52]	*GBA*	TCCAGGTCGTTCTTCTGACT/ ATTGGGTGCGTAACTTTGTC
DA-specific gene expression (RT-qPCR)	*TH*	AAAGTGTCAGAGCTGGACAAG/ GAAGGCGATCTCAGCAATCA
	*LMX1A*	CTTGCATTCTTGCTCTCTTTGG/ CAGGAGTCTGGGCTTTACATT
	*NURR1*	CAGAGCTACAGTTACCACTCTTC/ TGGTGAGGTCCATGCTAAAC

## Data Availability

Clinical exome sequencing data are available at PRJNA563295, BioSample accession SAMN22788974, https://www.ncbi.nlm.nih.gov/biosample/22788974 (for GBA-PD PD30), and SAMN26587088, https://www.ncbi.nlm.nih.gov/biosample/26587088 (for GBA-carrier PD31) (all accessed on 17 February 2023). Characterization of iPSCs is presented in the Human Pluripotent Stem Cell Registry (hPSCreg): for GBA-PD (https://hpscreg.eu/cell-line/ICGi034-A; https://hpscreg.eu/cell-line/ICGi034-B; https://hpscreg.eu/cell-line/ICGi034-C; all accessed on 17 February 2023) and for GBA-carrier (https://hpscreg.eu/cell-line/ICGi039-A; https://hpscreg.eu/cell-line/ICGi039-B; https://hpscreg.eu/cell-line/ICGi039-C; all accessed on 17 February 2023).

## Supplementary Materials

The following supporting information can be downloaded at: https://www.mdpi.com/article/10.3390/ijms24054437/s1.
