# Supplementary material for: Biochemical Characteristics of iPSC-Derived Dopaminergic Neurons from N370S GBA Variant Carriers with and without Parkinson’s Disease
Source: Int J Mol Sci. 2023 Feb 23;24(5):4437. doi: 10.3390/ijms24054437 (PMC10002967; doi:10.3390/ijms24054437)
Supplement: Supplementary file 1 [file ijms-24-04437-s001.zip › ijms-2163006-supplementary.pdf]

Supplementary Materials

Obtaining and characterization of iPSCs from an individual carrying a mutation in the GBA gene

The methods used to obtain and characterize of iPSC lines in detail are described in [22].

The blood sampling of an individual carrying a mutation in the *GBA* gene was carried out in two 9 ml vacuum tubes with K3 EDTA (GreinerBio). Histopaque-1077 (Sigma) was used for isolation of peripheral blood mononuclear cells (PBMCs); isolation was performed according to the manufacturer's recommendations. PBMCs were frozen in 90% KnockOut Serum Replacement (Thermo Fisher Scientific), 10% DMSO (Sigma), 5-10 million cells per a cryovial.

iPSCs were generated according to standard methods for reprogramming patient-specific PBMCs. 1×10^6 of PBMCs were transfected on Neon Transfection System (Thermo Fisher Scientific), program: 1650 V, 10 ms, 3 times, using episomal vectors (OCT4, KLF4, L-MYC, SOX2, LIN28, and Trp53) (0.5 µg each; Addgene IDs #41855-58, #41813-14). PBMCs were thawed and cultured in StemPro34 SFM supplemented with 1% penicillin-streptomycin, 2 mM GlutaMax-I (all Thermo Fisher Scientific), 100 ng/ml SCF, 40 ng/ml IGF1, 25 ng/ml GM-CSF, 50 ng /ml IL-3, 3.6 ul/ml erythropoietin (all SCI Store), 1 µM dexamethasone 5 days before reprogramming. After transfection on the day of reprogramming, cells were planted on D35 mm Petri dishes with mitotically inactivated mouse embryonic fibroblasts (MEF) in the above medium without the addition of penicillin-streptomycin. Then, for the first 6 days, 500 µl of N2B27 medium, consisting of DMEM/F12, 1 M HEPES, 2 mM GlutaMax, 1% penicillin-streptomycin, 1% NEAA, 1x N-2 Supplement, 1x B-27 Supplement (all Thermo Fisher Scientific), 0.1 mM 2-mercaptoethanol (2-mce, Sigma) and 100 ng/ml rhFGF basic (bFGF, SCI Store) were added directly to the Petri dish every day. On day 7-8, the medium was completely changed to N2B27. From the 9th day of cultivation, the medium was completely replaced by iPSCs-medium containing KnockOut DMEM, 15% KnockOut Serum Replacement, GlutaMAX-I, 0.1 mM NEAA, 1% penicillin-streptomycin (all Thermo Fisher Scientific), 0.1 mM 2-mce (Sigma), and 10 ng/ml bFGF (SCI Store). On days 9-14 of cultivation, primary colonies appeared. The colonies were picked manually and transferred into individual drops of TrypLE Express (Thermo Fisher Scientific) for 5 minutes, pipetted, and the cell suspension was transferred to the wells of a 48-well plate with the MEF coated bottom.

iPSCs were passaged 2 times per a week at the ratio of 1:8 - 1:10 with the addition of 2 µg/ml ROCK inhibitor Thiazovivin (Sigma). TrypLE Express (Thermo Fisher Scientific) was used for passaging. The cells were cultured in CO_2 incubator (37°C, 5% CO2).

Three iPSC lines were selected after prolonged cultivation, characterized according to all requirements and registered in the Human Pluripotent Stem Cell Registry (hPSCreg, https://hpscreg.eu/) with accession numbers ICGi039-A, ICGi039-B and ICGi039-C.

All cell lines demonstrate an iPSC-like morphology (Figures S1A-S3A), have a large nuclear-cytoplasmic ratio, and intensively proliferate in dense monolayer colonies. To detect endogenous alkaline phosphatase by the histochemical method, the cells were fixed by drying in air and incubated in a dye solution: 100 µM Tris-HCl pH 9.0; 100 µM NaCl; 5 µM MgCl2; 0.4 µg/mL naphthol phosphate (Sigma); 1 µg/mL Fast Violet B Salt (Sigma), for 15–20 min in the dark at room temperature. All patient-specific iPSCs obtained were shown to express endogenous alkaline phosphatase (Figures S1B-S3B).

Karyotyping (G-banding) of ICGi039-A (20th passage), ICGi039-B (18th passage), ICGi039-C (17th passage) cell lines showed a normal chromosome set in each line (50 metaphase plates were analyzed) (Figures S1C-S3C). The cells were seeded by $2*10^5$ cells into 4 wells of a 12-well plate coated with the extracellular matrix Matrigel (Matrigel-GFR) for karyotyping. On the next day 0.05 μg/ml KaryoMAX Colcemid (Life Technologies) was added to the growth medium for 2.5 hours. Next, the cells were treated with TrypLE (Thermofisher Scientific) and immersed in the hypotonic solution of 0.28% KCl for 18–20 min at 37°C. The cell suspension was prefixed by adding 1–2 drops of a methanol-acetic acid (3:1) fixing mixture and centrifuged for 7 min at 1000g. The supernatant was removed, the cell pellet was resuspended in 1.5 ml of fixative and left for 15 min on ice. The cells were centrifuged and the fixative was changed twice. Metaphase chromosome preparations were analyzed in the Institute of Medical Genetics of the Tomsk National Research Medical Center.

For immunofluorescence analysis, iPSCs were fixed in 4% formaldehyde for 10 minutes, permeabilized in 0.5% Triton X-100 solution for 30 min, and incubated with blocking buffer (10 mg/ml BSA in PBS). The above procedures were carried out at room temperature. Incubation with primary antibodies was carried out overnight at +4°C. The cells were incubated with secondary antibodies in the dark at room temperature for 1-1.5 hours. Cell nuclei were stained with DAPI. Preparations were analyzed on the Nikon Ti fluorescence microscope using NIS Elements software. The list of primary and secondary antibodies is presented in Table S1. Immunofluorescence analysis showed expression of pluripotency markers such as transcription factors OCT4, SOX2 and surface markers SSEA-4 and TRA-1-60 (Figures S1D-3D).

The test for the ability of iPSCs to produce all three primary germ layers (ecto-, endo- and mesoderm) upon spontaneous differentiation was performed. iPSC cell colonies were detached from D35 mm Petri dishes using 0.15% type IV collagenase (Thermo Fisher Scientific) and transferred to Petri dishes (D35 mm) coated with 1% agarose in iPSC medium without bFGF supplemented with 2 μg/ml Thiazovivin. The cells were cultured for 9-10 days in suspension for embryoid body formation, and then the embryoid bodies were plated on Matrigel treated 8-well Chambered Coverglass plates (Thermo Fisher Scientific) and cultured for another 7-9 days. Next, the cells were fixed with 4% formaldehyde, and immunofluorescent staining was performed. The list of antibodies is given in Table S1. It is shown that the all three lines are able to differentiate into three germ layers: ectoderm (βIII Tubulin (TUBB/TUJ1)), endoderm (FOXA2 (or HNF3b), alpha-fetoprotein (AFP)) and mesoderm (alpha actinin smooth muscle (α-SMA), cardiac cell marker NKX2.5) (Figures S1E-S3E).

Quantitative real-time PCR was performed for all iPSC lines, demonstrating the presence of the expression of pluripotency markers OCT4, NANOG and SOX2, as well as in the human embryonic stem cell line, HUES9 (HVRDe009-A) (Figures S1F-S3F). RNA was isolated using Trizol (Thermo Fisher Scientific) for this assay. Reverse transcription of 1 μg RNA was performed using SuperScript III reverse transcriptase (Thermo Fisher Scientific). Quantitative PCR was performed on a LightCycler 480 II real-time PCR system (Roche) with a BioMaster HS-qPCR SYBR Blue 2× (Biolabmix) using the programs: 95°C 5 min; 40 cycles: 95◦C 10s, 60◦C 1 min. CT values were normalized to beta-2 microglobulin using the ΔΔCT method. The list of primers used is presented in Table S1.

To confirm the mutations, Sanger sequencing of the obtained iPSC lines, PBMCs of patients and a healthy control was performed. As a result of this test, the single nucleotide polymorphism rs76763715 (*c.1226A> G*, p.N370S), which was found in the *GBA* gene, was confirmed in iPSC lines (Figures S1G-S3G). The primers are listed in Table S1.

All lines were tested for the absence of mycoplasma contamination by PCR (95°C 5 min; 35 cycles: 95°C 15 s, 60°C 15 s, 72°C 20 s) on an S1000 Thermal Cycler (Bio-Rad). Primers are presented in Table S1.

Table S1. Summary table of antibodies and primers used to characterize patientspecific iPSCs.

	**Antibodies used for immunocytochemistry**			
	**Antibodies**	**Dilution**	**Company Cat #**	**RRID**
Pluripotency Markers	Rabbit IgG anti-OCT4	1:200	Abcam Cat # ab18976	RRID:AB_444714
	Mouse IgG3 anti-SSEA4	1:200	Abcam Cat # ab16287	RRID:AB_778073
	Mouse IgM anti-TRA-1-60	1:200	Abcam Cat # ab16288	RRID:AB_778563
	Rabbit IgG anti-SOX2	1:500	Cell Signaling Cat # 3579	RRID:AB_2195767
Differentiation Markers	Mouse IgG2a anti-αSMA	1:100	Dako Cat # M0851	RRID:AB_2223500
	Rabbit IgG anti-NKX2.5 (H-114)	1:100	Santa Cruz Biotechnology Cat # sc-14033	RRID: AB_650281
	Mouse IgG1 anti-HNF3b (FOXA2)	1:50	Santa Cruz Biotechnology Cat # sc-374,376	RRID:AB_10989742
	Mouse IgG2a anti-AFP	1:250	Sigma Cat # A8452	RRID: AB_258392
	Mouse IgG2a anti-Tubulin β 3 (TUBB3)/ Clone: TUJ1	1:1000	BioLegend Cat # 801201	RRID:AB_2313773
Secondary antibodies	Goat anti-Mouse IgG (H + L) Secondary Antibody, Alexa Fluor 488	1:400	Thermo Fisher Scientific Cat # A11029	RRID:AB_2534088
	Goat anti-Mouse IgG (H + L) Secondary Antibody, Alexa Fluor 568	1:400	Thermo Fisher Scientific Cat # A11031	RRID:AB_144696

	Goat anti-Rabbit IgG (H + L) Highly Cross-Adsorbed Secondary Antibody, Alexa Fluor 488	1:400	Thermo Fisher Scientific Cat # A11008	RRID:AB_143165
	Goat anti-Rabbit IgG (H + L) Alexa Fluor 568	1:400	Thermo Fisher Scientific Cat # A11011	RRID:AB_143157
	Goat anti-Mouse IgG1 Alexa Fluor 568	1:400	Thermo Fisher Scientific Cat # A21124	RRID: AB_2535766
	Goat anti-Mouse IgG2a Alexa Fluor 488	1:400	Thermo Fisher Scientific Cat # A21131	RRID: AB_2535771
	**Primers**			
	**Target**	**Size**	**Forward/Reverse primer (5′-3′)**	
House-keeping gene (RT-qPCR)	beta-2-microglobulin	280 bp	TAGCTGTGCTCGCGCTACT/TCTCTGCTGGATGACGTGAG	
Pluripotency marker (RT-qPCR)	*NANOG*	391 bp	CAGCCCCGATTCTTCCACCAGTCCC/CGGAAGATTCCCAGTCGGGTTCACC	
	OCT4	94 bp	CTTCTGCTTCAGGAGCTTGG/GAAGGAGAAGCTGGAGCAAA	
	SOX2	100 bp	GCTTAGCCTCGTCGATGAAC/AACCCCAAGATGCACAACTC	
Targeted mutation analysis	*GBA*	600 bp	CTGTTGCTACCTAGTCACTTCC/CCCTATCTTCCCTTTCCTTCAC	
Mycoplasma detection	16S ribosomal RNA gene	280 bp	GGGAGCAAACAGGATTAGATACCCT/TGCACCATCTGTCACTCTGTTAACCTC	

Table S2. Statistics of known SNVs revealed in GBA-PD patient (PD30) and GBA-carrier (PD31) after clinical exome data analysis.

KNOWN VARIANTS	PD30	PD31
DBSNP	14,913(99.79%)	14,839(99.77%)

1000 GENOMES	14,698(98.35%)	14,632(98.38%)
GNOMAD	14,900(99.7%)	14,828(99.7%)
CLINVAR	8,466(56.65%)	8,545(57.45%)
Pathogenic	8(0.05%)	8(0.05%)
Likely pathogenic	8(0.05%)	5(0.03%)
Uncertain significance	108(0.72%)	102(0.69%)
Likely benign	474(3.17%)	497(3.34%)
Benign	8,292(55.48%)	8,376(56.32%)
Affects	4(0.03%)	2(0.01%)
Association	4(0.03%)	2(0.01%)
Confers sensitivity	2(0.01%)	0
Conflicting interpretations of pathogenicity	94(0.63%)	87(0.58%)
Drug response	22(0.15%)	22(0.15%)
Established risk allele	1(0.01%)	1(0.01%)
Likely risk allele	1(0.01%)	2(0.01%)
No interpretation for the single variant	4(0.03%)	3(0.02%)
Not provided	4(0.03%)	7(0.05%)
Other	3(0.02%)	6(0.04%)
Protective	2(0.01%)	1(0.01%)
Risk factor	12(0.08%)	8(0.05%)
DBNSFP	2,869(19.2%)	2,790(18.76%)

Table S3. Clinically significant SNVs revealed in GBA-PD patient (PD30) and GBA-carrier (PD31) after clinical exome data analysis.

Locus	HGVSp	HGVSc	Exon	PD30 Depth (Alt/Ref)	PD31 Depth (Alt/Ref)	dbSNP reference	gnomAD AF	ClinVar: Clinical significance
GBA	p.N370S	c.1226A> G	9	138 (68/70 51%)	181 (81/98 54%)	rs76763715	2.311e-3	Pathogenic
MTHFR	p.E429A	c.1286A>C	8	156 (68/88 44%)	210 (94/116 45%)	rs1801131	0.263	Likely pathogenic
LRP8	p.R952Q	c.2855G>A	19	76 (34/42 45%)	55 (0/55 0%)	rs5174	0.277	Risk factor
ABCA4	p.R943Q	c.2828G>A	19	61 (31/30 51%)	107 (50/57 47%)	rs1801581	0.029	Likely pathogenic
AMPD1	p.K320I	c.959A>T	7	47 (29/18 62%)	44 (0/44 0%)	rs34526199	0.026	Likely pathogenic
AMPD1	p.Q45X	c.133C>T	2	55 (0/55 0%)	102 (57/45 56%)	rs17602729	0.085	Pathogenic
SPTA1		c.6531-12C>T		61 (32/29 52%)	90 (90/0 100%)	rs28525570	0.254	Pathogenic
SPTA1	p.L1858V	c.5572C>G	40	47 (22/25 47%)	95 (95/0 100%)	rs3737515	0.254	Pathogenic
RNASEL	p.R462Q	c.1385G>A	2	129 (54/75 42%)	89 (0/89 0%)	rs486907	0.282	Risk factor
KLF11	p.Q62R	c.185A>G	2	93 (0/93 0%)	191 (97/94 51%)	rs35927125	0.088	Likely pathogenic
BTD	p.D424H	c.1270G>C	4	123 (55/68 45%)	116 (0/116 0%)	rs13078881	0.029	Pathogenic
ADH1B		c.18+13026A>G		67 (31/36 46%)	107 (56/51 52%)	rs698	0.313	Protective
ADH1C	p.I350V	c.1048A>G	8	67 (31/36 46%)	107 (56/51 52%)	rs698	0.313	Protective
IL13	p.Q144R	c.431A>G	4	99 (50/49 51%)	157 (85/72 54%)	rs20541	0.77	Risk factor
FBXO38	p.R526Q	c.1577G>A	12	44 (19/25 43%)	70 (0/70 0%)	rs376255193	3.945e-5	Likely pathogenic
HFE	p.H63D	c.187C>G	2	99 (54/45 55%)	159 (77/82 48%)	rs1799945	0.102	Pathogenic
CYP21A2		c.293-13C>A		44 (44/0 100%)	53 (45/8 85%)	rs6467	0.609	Pathogenic
HLA-DPB1	p.K98E	c.292A>G	2	128 (57/71 45%)	201 (200/1 100%)	rs1042140	0.243	Risk factor
ENPP1	p.K173Q	c.517A>C	4	60 (30/30 50%)	79 (41/38 52%)	rs1044498	0.328	Likely pathogenic

SOD2	p.V16A	c.47T>C	2	40 (40/0 100%)	72 (34/38 47%)	rs4880	0.47	Risk factor
NPSR1	p.N107I	c.320A>T	3	75 (41/34 55%)	94 (49/45 52%)	rs324981	0.472	Risk factor
STOX1	p.Y153H	c.457T>C	2	52 (16/36 31%)	67 (67/0 100%)	rs1341667	0.578	Risk factor
DRD4	p.A79SfsX21	c.235_247del	1	43 (0/43 0%)	130 (63/67 48%)	rs587776842	0.01	Pathogenic
ACTN3	p.R577X	c.1729C>T	15	63 (30/33 48%)	65 (0/65 0%)	rs1815739	0.375	Affects, Pathogenic
ATM		c.497-1G>A		33 (6/27 18%)	57 (0/57 0%)	rs778624615	6.874e-6	Likely pathogenic
OLR1	p.K167N	c.501G>C	4	121 (60/61 50%)	93 (0/93 0%)	rs11053646	0.126	Risk factor
GALC		c.1162-4del		65 (63/2 97%)	73 (69/2 95%)	rs11300320	0.959	Likely pathogenic
XRCC3		c.562-14A>G		60 (0/60 0%)	97 (53/44 55%)	rs1799796	0.281	Risk factor
KLC1		c.1849-1065T>C		60 (0/60 0%)	97 (53/44 55%)	rs1799796	0.281	Risk factor
LOXL1	p.G153D	c.458G>A	1	239 (121/118 51%)	37 (0/37 0%)	rs3825942	0.222	Risk factor
CHRNA3	p.Y215=	c.645C>T	5	187 (98/89 52%)	271 (271/0 100%)	rs1051730	0.258	Risk factor
IL4R	p.S503P	c.1507T>C	11	201 (76/125 38%)	79 (0/79 0%)	rs1805015	0.217	Protective
IL4R	p.Q576R	c.1727A>G	11	217 (97/120 45%)	79 (0/79 0%)	rs1801275	0.347	Risk factor
NQO1	p.P187S	c.559C>T	6	52 (24/28 46%)	73 (31/42 42%)	rs1800566	0.216	Pathogenic
MC1R	p.R160W	c.478C>T	1	230 (106/124 46%)	379 (182/197 48%)	rs1805008	0.046	Pathogenic
CLDN14	p.W30X	c.89G>A	2	63 (0/63 0%)	174 (91/83 52%)	rs1273842424	2.628e-5	Likely pathogenic

Table S4. SNVs in PD-associated genes revealed in GBA-patient (PD30) and GBA-carrier (PD31) after clinical exome data analysis.

Locus	HGVSp	HGVSc	Exon	PD30 Depth (Alt/Ref)	PD31 Depth (Alt/Ref)	dbSNP reference	gnomAD AF	ClinVar
GBA	p.N370S	c.1226A> G	9	138 (68/70 51%)	181 (81/98 54%)	rs76763715	2.311e-3	Pathogenic
PINK1		c.960-5G>A		79 (79/0 100%)	88 (88/0 100%)	rs3131713	0.842	Benign
PINK1-AS		n.3981+30C>T		79 (79/0 100%)	88 (88/0 100%)	rs3131713	0.842	Benign
PINK1	p.N521T	c.1562A>C	8	46 (0/46 0%)	180 (89/91 49%)	rs1043424	0.277	Likely benign
PINK1-AS		n.1687T>G	1	46 (0/46 0%)	180 (89/91 49%)	rs1043424	0.277	Likely benign
DDOST	p.G105=	c.315C>T	3	68 (68/0 100%)	117 (116/1 99%)	rs4704	0.626	Benign
DNAJC6		c.544-9C>T		78 (38/40 49%)	93 (0/93 0%)	rs2296481	0.252	Benign
DNAJC6	p.H502=	c.1506C>T	12	43 (43/0 100%)	85 (41/44 48%)	rs11208644	0.179	Benign
DNAJC6	p.S661=	c.1983T>C	13	85 (85/0 100%)	140 (63/77 45%)	rs12077111	0.179	Benign
DNAJC6	p.P670=	c.2010A>C	13	82 (81/1 99%)	147 (65/82 44%)	rs4582839	0.693	Benign
DNAJC6	p.S728N	c.2183G>A	15	69 (69/0 100%)	137 (64/73 47%)	rs4915691	0.208	Benign
GIGYF2	p.E518=	c.1554G>A	14	43 (0/43 0%)	81 (39/42 48%)	rs2305138	0.079	Benign
GIGYF2	p.Q980=	c.2940A>G	24	111 (53/58 48%)	172 (67/105 39%)	rs3816334	0.683	Benign
EIF4G1	p.M432V	c.1294A>G	10	170 (169/1 99%)	227 (227/0 100%)	rs2178403	0.799	Benign
EIF4G1		c.3953+9A>G		98 (98/0 100%)	146 (146/0 100%)	rs939317	0.795	Benign
SLC6A3		c.*35T>C	15	140 (68/72 49%)	260 (112/148 43%)	rs1042098	0.305	Benign
SLC6A3	p.S405=	c.1215A>G	9	79 (42/37 53%)	87 (0/87 0%)	rs6347	0.317	Benign
SLC6A3		c.1031+71G>T		100 (100/0 100%)	168 (168/0 100%)	rs40358	0.806	Benign
SLC6A3		c.419-12C>A		94 (38/56 40%)	61 (0/61 0%)	rs460000	0.311	Benign
PRKAG2		c.*112A>G	16	71 (39/32 55%)	84 (0/84 0%)	rs8961	0.55	Benign
PRKAG2		c.-26C>T	1	95 (0/95 0%)	233 (117/116 50%)	rs66628686	0.147	Benign
SLC18A2		c.791-42C>A		101 (101/0 100%)	164 (164/0 100%)	rs363343	0.741	Benign
SLC18A2		c.1306+43G>A		107 (107/0 100%)	157 (157/0 100%)	rs363272	0.802	Benign
LRRK2	p.R50H	c.149G>A	1	36 (36/0 100%)	69 (69/0 100%)	rs2256408	0.972	Benign
LRRK2	p.L153=	c.457T>C	5	47 (29/18 62%)	71 (23/48 32%)	rs10878245	0.509	Benign
LRRK2		c.1102-10C>A		42 (17/25 40%)	68 (41/27 60%)	rs7955902	0.326	Benign
LRRK2	p.L953=	c.2857T>C	22	56 (30/26 54%)	47 (0/47 0%)	rs7966550	0.092	Benign
LRRK2	p.P1542S	c.4624C>T	32	38 (0/38 0%)	77 (33/44 43%)	rs33958906	0.03	Benign

LRRK2	p.G1624=	c.4872C>A	34	77 (25/52 32%)	99 (99/0 100%)	rs1427263	0.698	Benign
LRRK2	p.K1637=	c.4911A>G	34	78 (26/52 33%)	107 (55/52 51%)	rs11176013	0.567	Benign
LRRK2	p.S1647T	c.4939T>A	34	82 (29/53 35%)	108 (51/57 47%)	rs11564148	0.272	Benign
LRRK2	p.E2108=	c.6324G>A	43	56 (20/36 36%)	65 (23/42 35%)	rs10878405	0.272	Benign
LRRK2	p.G2385=	c.7155A>G	48	39 (0/39 0%)	109 (48/61 44%)	rs33962975	0.109	Benign
LRRK2	p.M2397T	c.7190T>C	49	22 (11/11 50%)	44 (44/0 100%)	rs3761863	0.616	Benign
VPS35	p.H646=	c.1938C>T	15	95 (95/0 100%)	122 (122/0 100%)	rs168745	0.977	Benign
VPS35		c.915-3del		70 (17/40 24%)	97 (19/63 20%)	rs569369937	3.144e-3	Benign
ORC6		c.-20T>C	1	89 (0/89 0%)	234 (106/128 45%)	rs33994299	0.334	Benign
NDUFV2	p.V29A	c.86T>C	2	48 (48/0 100%)	84 (84/0 100%)	rs906807	0.79	Benign
SYNJ1		c.*186_*187insAATACT	33	127 (127/0 100%)	195 (87/108 45%)	rs57257560	0.553	Benign
SYNJ1		c.3588+14C>T		85 (36/49 42%)	109 (53/56 49%)	rs11702774	0.174	Benign
SYNJ1		c.3518-7G>T		86 (86/0 100%)	125 (64/61 51%)	rs2833929	0.283	Benign
SYNJ1	p.K295R	c.884A>G	8	91 (91/0 100%)	139 (63/76 45%)	rs2254562	0.292	Benign
FBXO7		c.122+272T>G		98 (59/39 60%)	104 (0/104 0%)	rs8137714	0.189	Benign
FBXO7	p.M115I	c.345G>A	2	108 (54/54 50%)	77 (0/77 0%)	rs11107	0.416	Benign
FBXO7	p.L317=	c.949C>T	6	57 (28/29 49%)	49 (0/49 0%)	rs9726	0.416	Benign

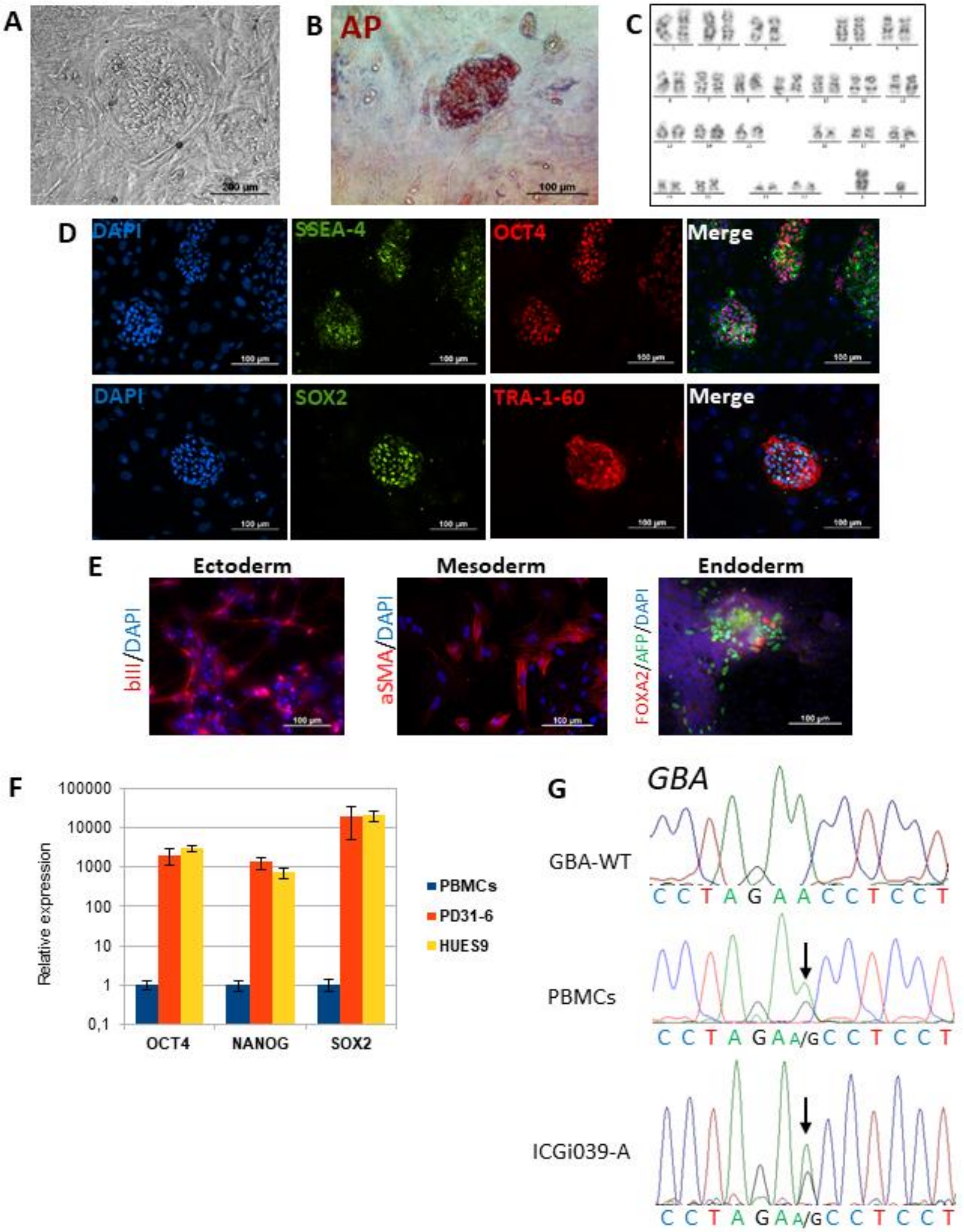

Figure S1. Characteristics of the IPSC ICGi039-A (PD31-6) line. **A.** Morphology of iPSC colonies. **B.** Histochemical detection of alkaline phosphatase (AP). **C.** Karyotype PD31-6, passage 20 (G-banding). **D.** Immunofluorescent staining for pluripotency markers: OCT4 (red), SOX2 (green), SSEA4 (green), TRA-1-60 (red). **E.** Immunofluorescent staining for markers of three germ layers: ectoderm (bIII-tubulin (red)), mesoderm (aSMA (red)) and endoderm (FOXA2 (red), AFP (green)). Nuclei are stained with DAPI (blue). **F.** Real-time PCR of pluripotency markers (OCT4, NANOG, SOX2) of ICGi039-A iPSC line, patient PBMCs and HUES9 ES cell line. **G.** Sequences of the GBA gene regions of PBMCs of a patient with PD, iPSC line ICGi039-A, and a healthy donor (control). Detected polymorphisms are marked with arrows.

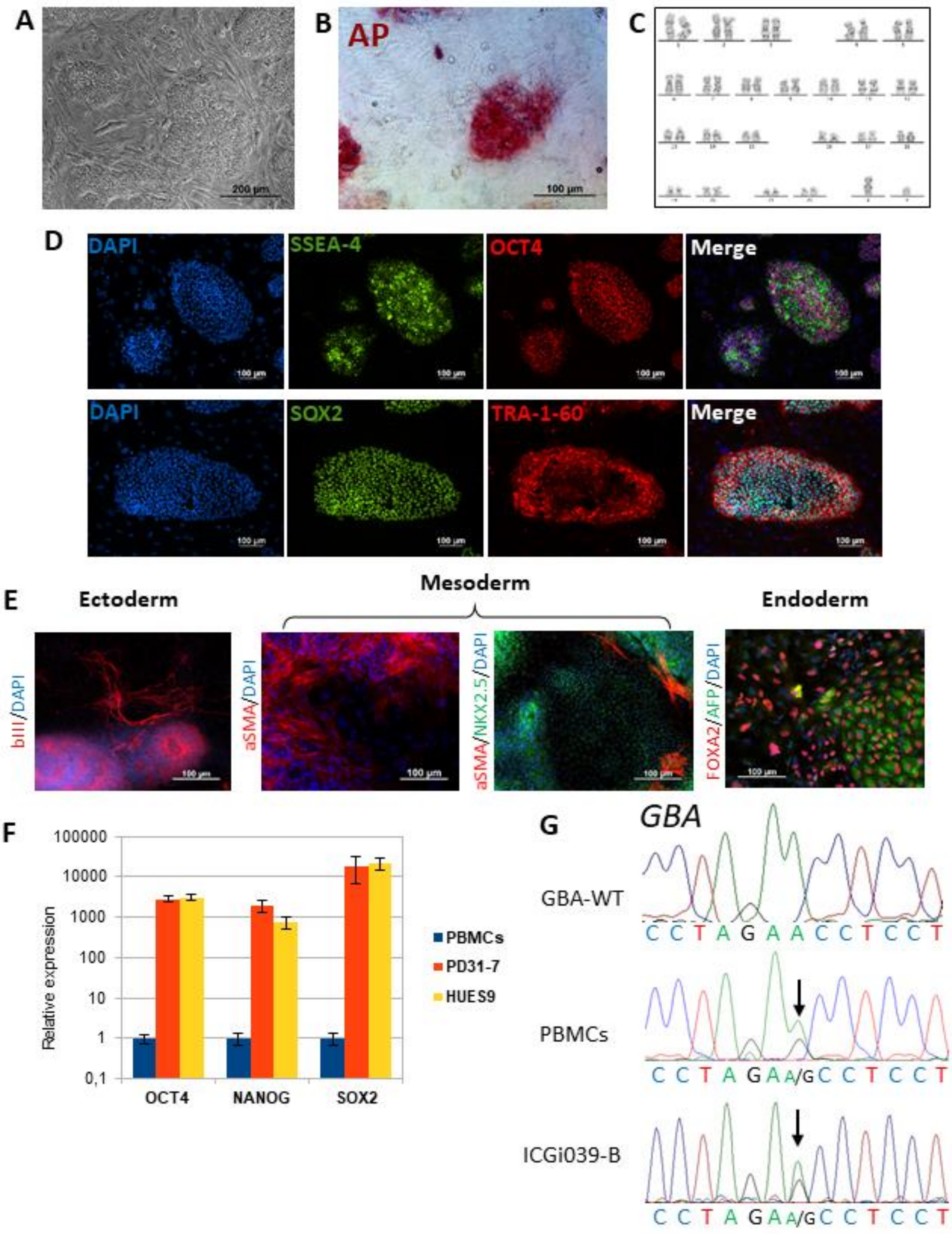

Figure S2. Characteristics of the IPSC ICGi039-B (PD31-7) line. **A.** Morphology of iPSC colonies. **B.** Histochemical detection of alkaline phosphatase (AP). **C.** Karyotype PD31-6, passage 18 (G-banding). **D.** Immunofluorescent staining for pluripotency markers: OCT4 (red), SOX2 (green), SSEA4 (green), TRA-1-60 (red). **E.** Immunofluorescent staining for markers of three germ layers: ectoderm (bIII-tubulin (red)), mesoderm (aSMA (red), NKX2.5 (green)) and endoderm (FOXA2 (red), AFP (green)). Nuclei are stained with DAPI (blue). **F.** Real-time PCR of pluripotency markers (OCT4, NANOG, SOX2) of ICGi039-B iPSC line, patient PBMCs and HUES9 ES cell line. **G.** Sequences of the GBA gene regions of PBMCs of a patient with PD, iPSC line ICGi039-B, and a healthy donor (control). Detected polymorphisms are marked with arrows.

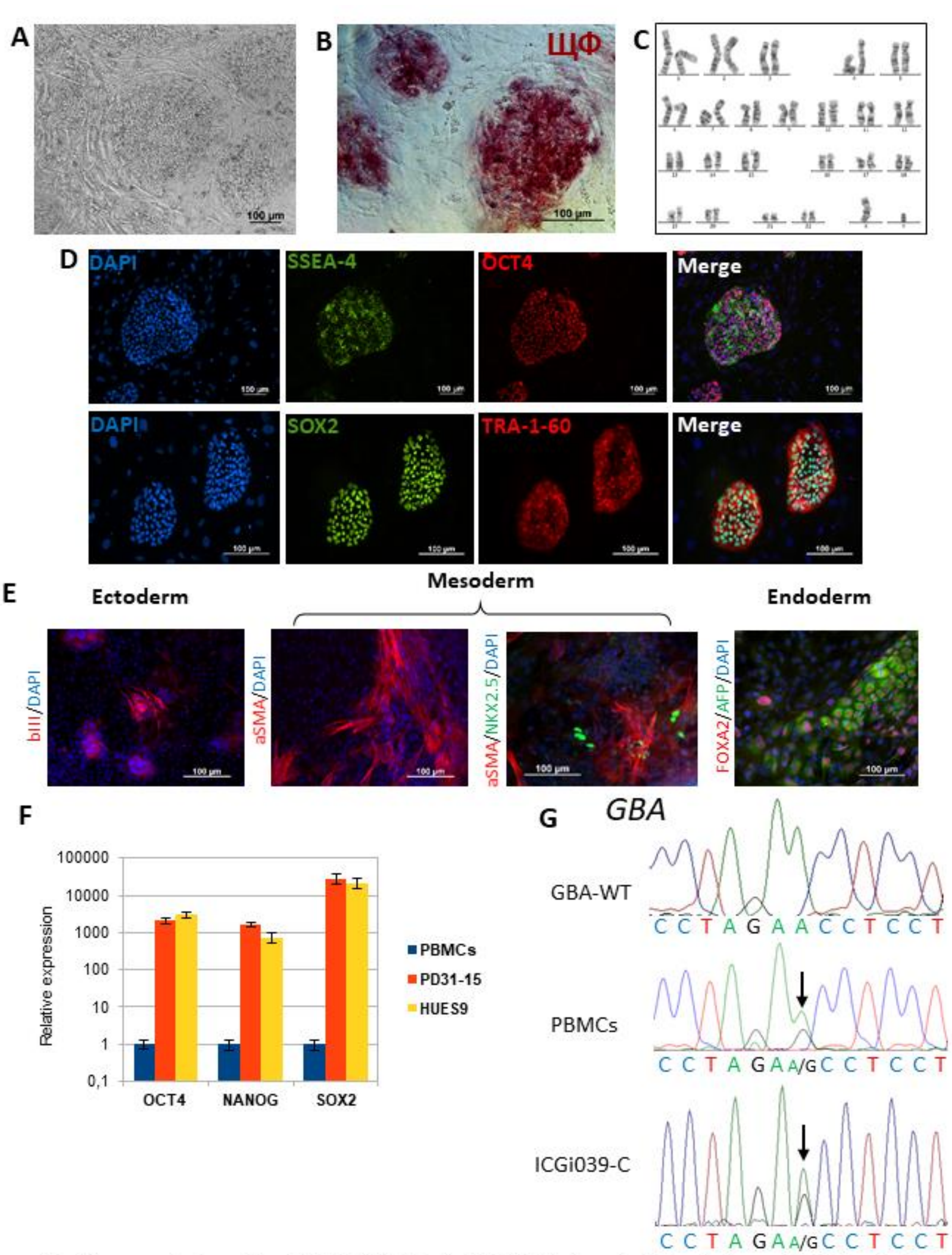

Figure S3. Characteristics of the IPSC ICGi039-C (PD31-15) line. **A.** Morphology of iPSC colonies. **B.** Histochemical detection of alkaline phosphatase (AP). **C.** Karyotype PD31-15, passage 17 (G-banding). **D**. Immunofluorescent staining for pluripotency markers: OCT4 (red), SOX2 (green), SSEA4 (green), TRA-1-60 (red). **E.** Immunofluorescent staining for markers of three germ layers: ectoderm (bIII-tubulin (red)), mesoderm (aSMA (red), NKX2.5 (green)) and endoderm (FOXA2 (red), AFP (green)). Nuclei are stained with DAPI (blue). **F.** Real-time PCR of pluripotency markers (OCT4, NANOG, SOX2) of ICGi039-C iPSC line, patient PBMCs and HUES9 ES cell line. **G.** Sequences of the GBA gene regions of PBMCs of a patient with PD, iPSC line ICGi039-C, and a healthy donor (control). Detected polymorphisms are marked with arrows.

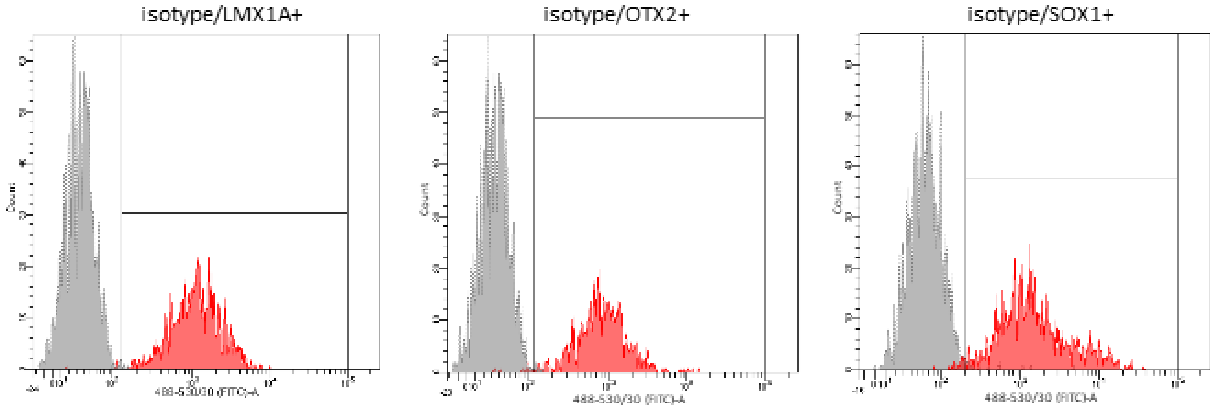

Figure S4. Flow cytometry of DA neurons progenitors. Results of flow cytometry of LMX1A-, OTX2-, SOX1-positive cells using one sample as an example. Isotype control labeled with secondary antibodies (grey), sample (red).

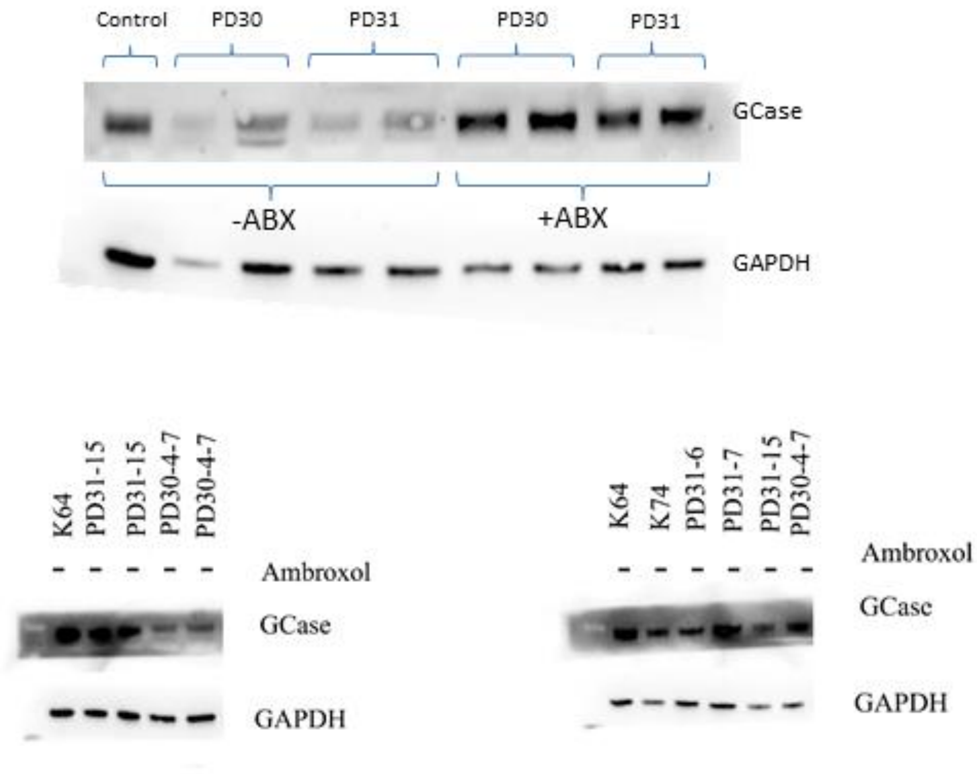

Figure S5. Western blot analysis of proteins before and after co-cultivation with ambroxol.
